# Three-Dimensional Surgical Guides in Orthodontics: The Present and the Future

**DOI:** 10.3390/dj13020074

**Published:** 2025-02-08

**Authors:** Silvia Izabella Pop, Eugen Bud, Kinga Mária Jánosi, Anamaria Bud, Bernadette Kerekes-Máthé

**Affiliations:** Faculty of Dental Medicine, George Emil Palade University of Medicine, Pharmacy, Science, and Technology of Targu Mures, 38 Gh. Marinescu Str., 540139 Târgu Mureș, Romania; silvia.pop@umfst.ro (S.I.P.); kinga.janosi@umfst.ro (K.M.J.); anamaria.bud@umfst.ro (A.B.); bernadette.kerekes-mathe@umfst.ro (B.K.-M.)

**Keywords:** orthodontics, surgical guides, 3D printing

## Abstract

Surgical guides are integral tools in orthodontics, enhancing the precision and predictability of mini-implant placement. These guides facilitate accurate positioning, reduce risks to surrounding anatomical structures, and ensure proper angulation and depth during procedures. The aim of the present paper is to present a detailed review of the surgical guides used in orthodontics, focusing on their classification, mechanical properties, biocompatibility, and future developments. The advantages, disadvantages, clinical steps, and implications are also described based on the data in recent scientific literature. Future developments may incorporate artificial intelligence and augmented reality, further optimizing treatment planning and patient outcomes, thus solidifying the role of surgical guides in efficient orthodontic care.

## 1. Introduction

Surgical guides have become an important tool in the placement of mini-implants in orthodontics, enhancing both the precision and predictability of the procedure [1]. These guides are designed to assist clinicians in accurately positioning mini-implants, which are increasingly utilized for anchorage in various orthodontic treatments. The primary properties of surgical guides include their ability to provide a predetermined path for implant insertion, reduce the risk of damaging surrounding anatomical structures, and facilitate the correct angulation and depth of implant placement [2].

Today, surgical guides are an essential component of orthodontic practice, incorporating advanced imaging techniques and biocompatible materials [3,4].

Current research in orthodontic surgical guides focuses on enhancing precision, customization, and integration with emerging technologies to improve patient outcomes. One significant area of study is the use of advanced imaging techniques, such as cone-beam computed tomography (CBCT), which provides detailed 3D images of a patient’s dental structures. A recent study by Chen et al. explores how CBCT data can be integrated with CAD software to design highly precise surgical guides that facilitate complex orthodontic procedures with minimal margins of error [5].

Researchers are also investigating the use of new materials and 3D printing methods to create more durable and biocompatible surgical guides. For instance, a study by Martinez et al. examines the use of novel photopolymer resins that can withstand sterilization processes without compromising their structural integrity, making them ideal for repeated clinical use [6].

There is ongoing research into the integration of digital workflows, where intraoral scanners, CAD/CAM systems, and 3D printers are seamlessly connected. This integration aims to streamline the production of surgical guides, reducing the turnaround time and allowing for real-time adjustments during orthodontic procedures, as highlighted by Lee and Kim [7].

The aim of the present paper is to present a detailed review of the surgical guides used in orthodontics focusing on their classification, mechanical properties, biocompatibility, and future developments.

## 2. Surgical Guides-Characteristics

### 2.1. History

The history of surgical guides in orthodontics, as detailed in the literature, illustrates the evolution of orthodontic practices and the increasing precision in treatment methodologies [8,9,10,11]. Initially, surgical guides were used for implant placement in edentulous patients [10,11]. Soon, orthodontists realized that guides can be helpful tools in mini-implant placements too. Orthodontic treatments were performed with limited precision tools, often relying on the skill and experience of the practitioner to achieve desired outcomes. However, as the field evolved, the need for more accurate and reliable methods became apparent, leading to the development of surgical guides [9].

In the early days, surgical guides were rudimentary, often handcrafted from wax or simple plastics to help align dental tools during procedures. According to a study by Graber et al., these early guides were limited in accuracy and relied heavily on the clinician’s skill [12]. The introduction of CAD/CAM (computer-aided design and computer-aided manufacturing) technology in the late 20th century marked a significant step forward. As noted by McNamara, this technology improved the ability to plan and execute orthodontic procedures with greater accuracy, significantly enhancing treatment outcomes [13].

By the early 2000s, 3D printing began to revolutionize the production of surgical guides. This technology enabled the rapid and cost-effective production of customized guides tailored to the unique anatomy of each patient. The use of biocompatible materials in 3D printing further enhanced the functionality and safety of these guides in clinical settings. A paper by Chen highlighted the benefits of 3D printing in creating highly customizable guides that could be tailored to the unique dental anatomy of each patient [14].

Today, surgical guides in orthodontics have become an integral part of treatment planning and execution, facilitating minimally invasive procedures and improving patient outcomes. The continuous advancements in digital imaging and 3D printing technologies promise to further enhance the precision and effectiveness of these tools, paving the way for more personalized and efficient orthodontic care.

### 2.2. Classification of Surgical Guides

Surgical guides used for mini-implant placement in orthodontics can be classified based on several factors:

1. Type of Mini-Implant: Guides can be tailored for specific types of mini-implants, such as threaded or plate-type implants.
2. Design Configuration: They can vary in design, including full-arch guides, segmental guides, or individualized guides that consider patient-specific anatomy.
3. Guiding Mechanism: Some guides use specific drilling protocols to ensure accurate placement, which can include stop mechanisms to control depth.
4. Manufacturing Process: They can be created using traditional methods (such as manual fabrication) or advanced digital techniques (like 3D printing) [15,16,17,18].

#### 2.2.1. Manufacturing

Several technologies are utilized in the fabrication of surgical guides for mini-implant placement, including:

1. CAD/CAM Technology: Computer-Aided Design (CAD) and Computer-Aided Manufacturing (CAM) allow for precise design and production of guides based on digital models. Subcategories of the CAD/CAM techniques include:

(a) 3D Printing: Additive manufacturing techniques enable the creation of complex guide shapes with high accuracy and customization for individual patient needs.

(b) Laser Cutting: Laser technology can be used to cut guides from sheets of biocompatible materials, ensuring precision and smooth edges.

2. Traditional Manual Fabrication: Although less common, some guides are still made using traditional methods, where the guide is crafted by hand based on the surgeon’s specifications [19,20,21,22].

Three-dimensional printing, or additive manufacturing procedures, include different technologies, according to the type of material and processing methods.

The most common 3D technologies, according to the literature are stereolithography (SLA), digital light processing (DLP), fused deposition modeling (FDM)/fused filament fabrication (FFF), selective laser sintering (SLS)/melting (SLM), electron beam melting (EBM), and binder jetting (BJ) [19]. While stereolithography (SLA) and digital light processing (DLP) technologies are preferred for their high accuracy, which is crucial for the precise fit and function of surgical guides, Selective Laser Sintering (SLS) and Material Jetting (MJ) offer unique advantages in terms of material strength and color resolution, which can be beneficial in certain clinical scenarios. SLA and DLP procedures use photopolymer resins that are cured layer by layer, ensuring detailed reproduction of the complex structures needed for orthodontic applications [22].

#### 2.2.2. Materials

Based on the common materials used for 3D printing, surgical guides can be classified as follows:

1. Photopolymer Resins: These are often used in stereolithography (SLA) 3D printing, providing high precision and detail.
2. Thermoplastic Polyurethane (TPU): Known for its flexibility and durability, TPU is suitable for guides that may require some adaptability during the surgical procedure.
3. Acrylic Resins: These materials are frequently used in various 3D printing methods and provide good mechanical properties along with biocompatibility.
4. Polyamide (Nylon): Commonly used in selective laser sintering (SLS), polyamide offers strength and biocompatibility, making it a popular choice for surgical guides [17,22,23,24].

### 2.3. Steps in Guide Fabrication

The primary function of surgical guides is to provide a physical template that directs surgical instruments or orthodontic tools during procedures. Traditionally, orthodontic treatments relied heavily on manual dexterity and experience, but the advent of digital technology has paved the way for more sophisticated surgical guides. These guides are typically created using advanced imaging techniques such as Cone Beam Computed Tomography (CBCT) or 3D scanning, which capture detailed anatomical data [25].

Once the data are collected, they are processed using specialized software, which allows orthodontists to plan the treatment meticulously. This digital model can then be used to fabricate a surgical guide using 3D printing technology. The resulting guide is a precise and patient-specific tool that enhances the accuracy of interventions, such as implant placement, ensuring they align perfectly with the planned treatment path.

**Figure 1:**
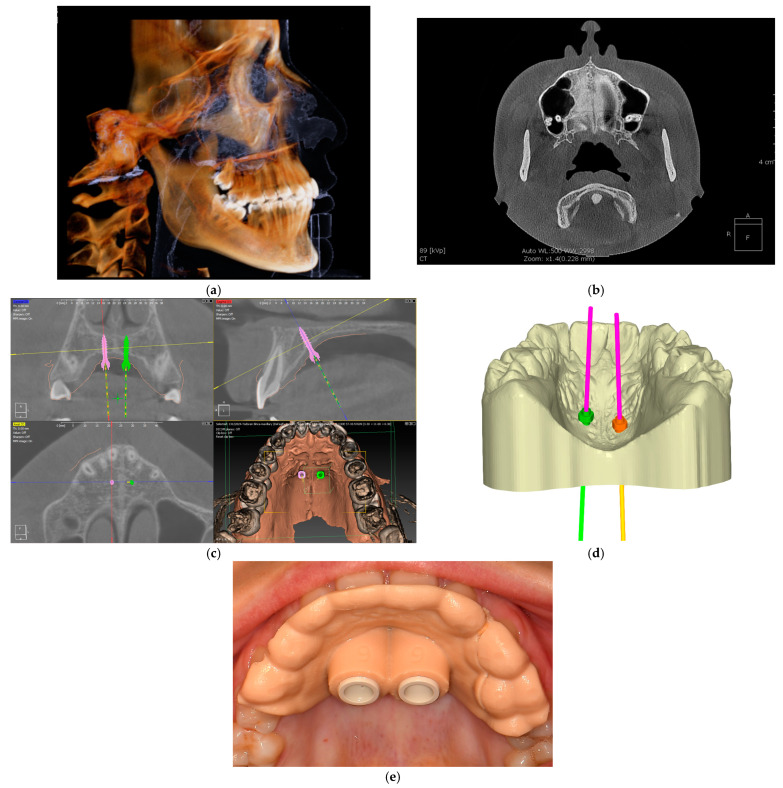
Steps for fabricating 3D surgical guides for orthodontics: (**a**) 3D image (cone beam computed tomography—CBCT) assessment of the patient 3D reconstruction of the cranium, (**b**) coronal view of the midpalatal suture, (**c**) digital planning of the mini-implants placement on the CBCT and (**d**) virtual model, (**e**) intraoral verification of the guide.

The steps for fabricating 3D surgical guides for orthodontics typically include (Figure 1a–e):

1. Patient Assessment and Imaging: Obtain detailed imaging data, usually through cone beam computed tomography (CBCT) and digital scans, to assess the patient’s anatomy and treatment needs.
2. Digital Model Creation: Using specialized software, create a 3D digital model of the patient’s dental structures based on imaging data.
3. Guide Design: Design the surgical guide in CAD software, ensuring it accommodates the specific surgical protocol and the type of mini-implants to be used. This design will include features for accurate drill alignment and depth control.
4. 3D Printing: Print the surgical guide using suitable 3D printing technology, such as SLA or FDM, ensuring that the material used is biocompatible and suitable for surgical applications.
5. Post-Processing: After printing, the guide may require cleaning, curing (if applicable), and sterilization to ensure it is safe for clinical use.
6. Clinical Verification: Prior to the surgical procedure, the guide should be verified in the patient’s mouth to ensure proper fit and alignment [19].

### 2.4. Advantages and Disadvantages of the Surgical Guides

The integration of digital technology in the creation of surgical guides offers numerous advantages. It allows for precise control over the placement of orthodontic appliances, ensuring they are positioned correctly according to the treatment plan. This precision reduces the risk of complications and enhances the overall effectiveness of the treatment. Moreover, digital workflows can significantly reduce the time required for planning and executing orthodontic procedures, leading to improved patient experiences [7,17,22,23,24,25,26,27].

However, despite their benefits, the use of surgical guides in orthodontics can present challenges. These include the need for specialized equipment and training, potential high costs, and the requirement for accurate digital impressions to ensure the guide’s effectiveness [26].

In terms of patient outcomes, surgical guides can significantly improve the results of orthodontic treatments. By ensuring that procedures are carried out with high precision, they contribute to better alignment, faster recovery times, and increased patient satisfaction. Patients often report feeling more confident in their treatment plan when visualizations and guides are used, as they provide a clear picture of the expected results [12,28].

#### 2.4.1. Advantages

The advantages of the surgical guides can be summarized as follows:

1. Precision and Accuracy
2. Customization
3. Reduction in Clinical Time
4. Patient Comfort [25,29].

The cost implications of using surgical guides in orthodontics can vary significantly depending on several factors, including the complexity of the case, the technology used, and the specific requirements of the treatment. Generally, surgical guides can increase the overall cost of orthodontic treatment due to the need for advanced imaging techniques, specialized software, and 3D printing technology [19,30].

One of the primary cost drivers is the initial investment in equipment and software required to create digital impressions and design the guides. Practices must invest in high-quality CBCT machines, 3D scanners, and 3D printers, which can be expensive. Additionally, the software used to process and design the guides often comes with licensing fees [27,31].

Another cost consideration is the production of the guides themselves. While 3D printing technology has become more accessible and cost-effective over time, the materials used for printing surgical guides must be biocompatible and of high quality, which can add to the expense [32].

#### 2.4.2. Disadvantages

The disadvantages of the surgical guides can be summarized as follows:

1. Cost
2. Longer learning curve
3. Specific technical laboratory requirements
4. Health risk related to the eventual toxicity of 3D printing [30,31,32].

### 2.5. Mechanical Properties of Surgical Guides

The main mechanical properties of surgical guides used in orthodontics are critical in ensuring their effectiveness and reliability during procedures. Flexural and tensile tests are recommended to evaluate the mechanical properties of materials used for surgical guide manufacturing. Standard procedures for assessing flexural and tensile properties recommend testing a minimum of five specimens for each sample, particularly for isotropic materials or molded specimens [25].

**Figure 2:**
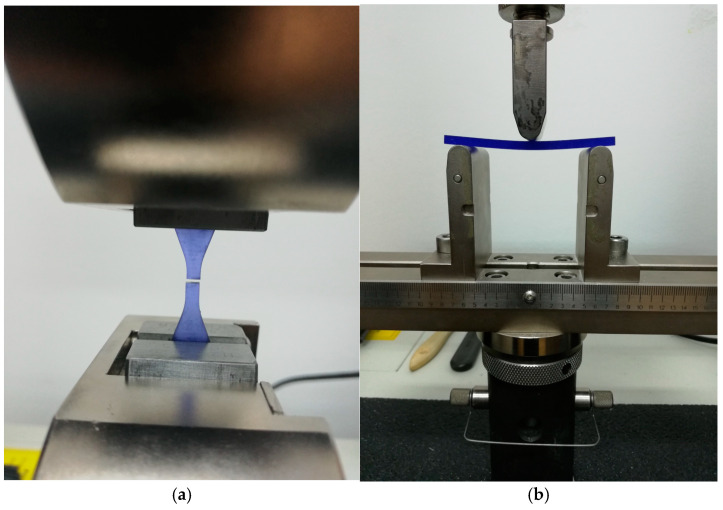
Tensile (**a**) and flexural (**b**) tests used for evaluation of the mechanical properties of standard specimens. The specimens are made of the same materials as the surgical guides but have standard geometrical forms.

The specimens used for the tensile and flexural tests (Figure 2a,b) are produced following specific standards (ASTM D638-14 and ASTM D790-03) [33,34]. In contrast to standard specimens, which are flat, surgical guides possess a unique geometrical morphology with a convex shape designed to fit the maxillary palatum. Quintana [35] indicated that the mechanical properties of photo-curable resins used in SLA printing are affected by the build orientations (flat or edge) of the components. Kazemi [36] observed that the presence of supporting structures can influence the tensile strength of parts fabricated through stereolithography (SLA) by increasing the surface roughness of the samples.

Studies [37,38,39,40,41] have identified several key properties that contribute to their performance:

1. Strength and Durability.

Surgical guides must exhibit sufficient strength to withstand the mechanical forces applied during drilling or appliance placement. Research by van Noort (2012) highlights that robust materials, such as polyetheretherketone (PEEK) and titanium, are often used due to their excellent mechanical properties, including high tensile strength and resistance to wear and fatigue [42].

2. Rigidity and Flexibility.

A study by Mangano et al. (2018) emphasizes that rigid guides prevent the deviation of surgical instruments, thereby enhancing procedural accuracy. The rigidity of a guide is often achieved using polymers or composites designed to maintain shape under stress [43]. While rigidity is important, some guides are designed with slight flexibility to accommodate minor anatomical variations without affecting performance. This balance between rigidity and flexibility is crucial for adapting to the unique contours of a patient’s anatomy [35].

3. Precision and Fit.

The guide must precisely fit the patient’s dental anatomy to function correctly. Precision is achieved through accurate digital modeling and 3D printing techniques, as discussed by Alharbi et al. (2016). This ensures that the guide aligns perfectly with the planned surgical path, reducing errors and improving outcomes [44].

4. Stability.

Stability is essential to ensure that the guide maintains its properties after sterilization and disinfection.

The mechanical properties of surgical guides are vital for their function. These properties are continually enhanced through research and advancements in materials science and manufacturing technologies.

### 2.6. Biocompatibility and Sterilization

Since surgical guides come into contact with the patient’s oral tissues, the materials used must be biocompatible to avoid any adverse reactions or allergies. This means that the materials should not provoke immune responses and should be safe for use within the human body. Common materials include medical-grade plastics and resins that have been tested for biocompatibility. The literature on the biocompatibility of surgical guides indicates several important findings. Research by Burbano et al. (2020) investigated different acrylic resins, demonstrating that modifications in their formulation can enhance biocompatibility and reduce cytotoxic effects [45]. A study by Shi et al. (2021) assessed the biocompatibility of various 3D-printed materials, such as biocompatible resins, concluding that they exhibit good cell viability and tissue integration [46].

A systematic review concluded that the use of biocompatible surgical guides leads to lower rates of postoperative complications and improved integration of implants, further supporting their importance in clinical practice [47].

Autoclave sterilization is a widely recognized and effective method for sterilizing surgical guides, ensuring they are free from any viable microorganisms before use in surgical procedures. Sterilization refers to the complete elimination of all microbial life through physical or chemical processes, while disinfection targets the removal of microorganisms capable of forming bacterial spores [48]. According to standard ISO 17664, manufacturers must provide information on the recommended disinfection and sterilization methods for the materials used in medical devices in the material’s data sheet [49,50]. Many suppliers of 3D printing materials recommend chemical disinfection using 70% isopropyl alcohol and autoclave sterilization, making it essential to examine how these methods impact the mechanical properties of the materials and their performance during clinical use.

According to Rutala (2016), the use of steam sterilization, such as that performed in an autoclave, is one of the most reliable methods for achieving sterilization in healthcare settings, including dental and orthopedic surgeries where surgical guides are commonly used [51].

A 2021 study by Smith et al. highlighted the effectiveness of autoclave sterilization in deactivating a broad spectrum of pathogens, emphasizing its role in maintaining high standards of infection control in surgical environments. This study confirmed that surgical guides made from common materials, such as polyetheretherketone (PEEK) and titanium, can withstand the autoclave’s high temperatures without compromising structural integrity [52].

A 2023 investigation by Martinez et al. explored the interplay between autoclave settings and sterilization efficiency for complex surgical guide designs. Their findings underscored the necessity of customizing autoclave parameters, such as cycle time and temperature, based on the guide’s material composition and design complexity to ensure thorough sterilization [53].

In 2023, a study by Zhao et al. focused on the efficiency of different autoclave cycles, such as pre-vacuum and gravity displacement, on complex surgical guide designs. The research underscored the importance of selecting the appropriate cycle based on the guide’s material and design to ensure thorough sterilization without compromising the guide’s functionality [54].

These recent articles underscore the ongoing advancements in autoclave sterilization techniques and the continuous need to adapt these methods to accommodate new materials and technologies in surgical guide manufacturing.

The impact of sterilization on 3D-printed guides is a critical consideration in their clinical application, as the process can affect both the material properties and the dimensional accuracy of these guides. Recent studies [25,55] have focused on understanding these effects to ensure that 3D-printed surgical guides remain reliable and effective after undergoing sterilization.

A key concern is that the high temperatures and pressures used in autoclave sterilization can induce changes in the physical properties of certain 3D-printed materials. For instance, a study demonstrated that some common 3D printing materials, such as PLA and ABS, may experience slight warping or shrinkage after multiple autoclave cycles. This can potentially affect the fit and precision of surgical guides, which are critical for successful surgical outcomes [52].

Furthermore, research by Pop et al. (2022) explored the effect of autoclave sterilization on the mechanical properties of 3D-printed guides. They found that thermal sterilization produced an increase in the stiffness of all guides, and a higher sterilization temperature, leading to a stiffer guide [25].

Overall, understanding the impact of sterilization on 3D-printed guides is essential for ensuring their safety and effectiveness in clinical settings, and it highlights the need for continuous innovation in both materials science and sterilization technology.

### 2.7. Clinical Considerations

Clinical considerations regarding the use of surgical guides in orthodontics include several aspects. The accuracy of placement is one of the main issues. Surgical guides enhance the precision of mini-implant placement, which is critical for achieving desired orthodontic outcomes. Ensuring the guide is properly fitted and aligned with the planned implant sites is essential [4,5].

Individual anatomical variations must be considered when designing and using surgical guides. Preoperative imaging and assessment are crucial for creating a guide that accommodates the patient’s unique anatomical features [9].

The choice of biocompatible materials for the surgical guide is important, as it affects both the guide’s effectiveness and the patient’s healing process. Ensuring that the material can withstand sterilization methods is also key. The training and experience of the surgeon can impact the success of the procedure. Proper training in both the design and application of guides is vital for optimal outcomes [25].

### 2.8. Future of the Surgical Guides

The latest innovations in orthodontic guides reflect the rapid advancements in digital technology and materials science, aiming to enhance the precision, efficiency, and user-friendliness of orthodontic procedures. The literature continues to explore the potential of these technologies and investigates the role of artificial intelligence in further enhancing the precision and efficiency of orthodontic surgical guides [56,57,58]. AI-driven analysis significantly reduces the time needed for treatment planning and increases the accuracy of surgical guide fabrication [56].

The future enhancements in guided surgery in dentistry and orthodontics might be:

1. AI and Machine Learning Integration: Recent developments have seen the integration of artificial intelligence and machine learning algorithms in the design and planning phase of surgical guides. These technologies help in predicting optimal outcomes and refining treatment plans based on large datasets of previous cases, thus enhancing the customization and accuracy of guides [57].
2. Advanced 3D Printing Materials: Innovations in 3D printing materials have led to the development of more durable and biocompatible options. Materials such as resin composites and advanced polymers are being used to create guides that are not only strong and accurate but also safe for prolonged use in the oral environment [58].
3. Augmented Reality (AR) Applications: Augmented reality is being explored as a tool to enhance the planning and execution of orthodontic procedures. By overlaying digital information on the real-world view, AR can assist orthodontists in visualizing guide placement and ensuring precise alignment during surgery [57].
4. Cloud-Based Digital Workflows: The adoption of cloud-based platforms allows for seamless collaboration between orthodontists, dental technicians, and patients. These systems enable real-time sharing of digital models and treatment plans, facilitating more efficient and coordinated care [56].

The results of Lee’s study indicate that Virtual Surgical Plan VSP consistently reduces discrepancies between planned and actual surgical outcomes, particularly when integrated with custom surgical guides. Additionally, while VSP demonstrated potential time-saving advantages over conventional planning, these differences were not statistically significant across studies, likely due to high variability among study protocols and designs. As a conclusion, VSP with custom surgical guides enhances surgical accuracy in orthognathic procedures, marking a significant advancement over traditional methods [59].

Lee Y C in another study regarding the use of VSP shows a positive post-surgery impact on the quality of life of the patients, underlining the significant role of these technologies in enhancing self-esteem and reducing anxiety [60].

Althalhi in his article explores the role of AI in implant dentistry, emphasizing its impact on diagnostics, treatment planning, and patient outcomes. AI-driven image analysis and deep learning algorithms enhance the precision of implant placement, reducing risks and optimizing esthetics. Moreover, AI-driven data analytics provide valuable insights into patient-specific treatment strategies, improving overall success rates [61].

Macri, in a systematic review studying the results of the literature, indicates a growing interest in the application of AI in implant planning, with evidence suggesting an improvement in precision and predictability compared to traditional methods. The summary of the findings he obtained represents the latest AI developments in implant planning, demonstrating its application for the automated detection of bones, the maxillary sinus, neuronal structure, and teeth. Some disadvantages were also identified, including the need for high-quality training data and the lack of standardization in protocols. However, further research is needed to fully understand its potential and address the challenges associated with its implementation in clinical practice [62].

Satapathy included twenty patients requiring dental implants in a comparative study. For each patient, a clinical treatment plan was created by an experienced dentist, while an AI algorithm generated an alternative plan. Various parameters, including implant position, angulation, and depth, were compared between the two plans. Surgical templates were fabricated based on both plans to guide implant placement accurately. The AI-generated plan showed a reduction in planning time, averaging 10 min compared to the clinical planning, which averaged 30 min per case. Additionally, the surgical templates based on AI-generated plans exhibited similar accuracy in implant placement as those based on clinical plans. AI-assisted treatment planning for dental implant placement demonstrates promising results in terms of accuracy and efficiency [63].

These innovations are set to enhance the effectiveness of orthodontic guides, making them an even more valuable tool in achieving optimal treatment outcomes.

The fabrication of surgical guides for mini-implants shares foundational principles with those for classical dental implants, particularly in the utilization of imaging and computer-aided design (CAD) technologies. However, there are notable differences in protocol and materials due to the distinct dimensions and placement considerations of mini-implants. For instance, mini-implants often require a more simplified guide design due to their smaller size and the typically less invasive placement techniques involved. Additionally, the materials used for mini-implant guides may differ, as some practitioners prefer using lighter and more flexible materials like thermoplastics compared to the more rigid materials often employed for traditional implant guides [64]. These variations can affect the precision and ease of implant placement, highlighting the importance of tailored approaches for different implant types.

## 3. Conclusions

Surgical guides have become essential tools in orthodontics, particularly for the precise placement of mini-implants. Their development has transitioned from simple, handcrafted devices to advanced digital solutions, significantly improving treatment outcomes. Current research emphasizes enhanced precision, customization, and the integration of technologies like artificial intelligence (AI) and 3D printing. These innovations aim to optimize workflows, reduce planning times, and improve patient outcomes. Additionally, ongoing studies focus on developing biocompatible materials that ensure safety and effectiveness during procedures. As these technologies continue to advance, they promise to further enhance the role of surgical guides in delivering personalized and efficient orthodontic care.

## Data Availability

No new data were created or analyzed in this study. Data sharing is not applicable to this article.
